# Comparative Genomics of *Glossina palpalis gambiensis* and *G. morsitans morsitans* to Reveal Gene Orthologs Involved in Infection by *Trypanosoma brucei gambiense*

**DOI:** 10.3389/fmicb.2017.00540

**Published:** 2017-04-03

**Authors:** Illiassou Hamidou Soumana, Bernadette Tchicaya, Stéphanie Rialle, Hugues Parrinello, Anne Geiger

**Affiliations:** ^1^UMR 177, Institut de Recherche pour le Développement-CIRAD, CIRAD TA A-17/GMontpellier, France; ^2^Centre National de la Recherche Scientifique Unité Mixte de Recherche 5203, Institut de Génomique FonctionnelleMontpellier, France; ^3^Institut National de la Santé Et de la Recherche Médicale U661Montpellier, France; ^4^Universités de Montpellier 1 and 2, UMR 5203Montpellier, France; ^5^Montpellier GenomiX, c/o Institut de Génomique FonctionnelleMontpellier, France

**Keywords:** human African Trypanosomiasis, *Glossina palpalis gambiensis*, *Glossina morsitans morsitans*, *Trypanosoma brucei gambiense*, differentially expressed genes, heterologous genes

## Abstract

Blood-feeding *Glossina palpalis gambiense* (Gpg) fly transmits the single-celled eukaryotic parasite *Trypanosoma brucei gambiense* (Tbg), the second *Glossina* fly African trypanosome pair being *Glossina morsitans*/*T*.brucei rhodesiense. Whatever the *T. brucei* subspecies, whereas the onset of their developmental program in the zoo-anthropophilic blood feeding flies does unfold in the fly midgut, its completion is taking place in the fly salivary gland where does emerge a low size metacyclic trypomastigote population displaying features that account for its establishment in mammals-human individuals included. Considering that the two *Glossina*—*T. brucei* pairs introduced above share similarity with respect to the developmental program of this African parasite, we were curious to map on the *Glossina morsitans morsitans* (Gmm), the Differentially Expressed Genes (DEGs) we listed in a previous study. Briefly, using the gut samples collected at days 3, 10, and 20 from Gpg that were fed or not at day 0 on Tbg—hosting mice, these DGE lists were obtained from RNA seq—based approaches. Here, post the mapping on the quality controlled DEGs on the Gmm genome, the identified ortholog genes were further annotated, the resulting datasets being compared. Around 50% of the Gpg DEGs were shown to have orthologs in the Gmm genome. Under one of the three *Glossina* midgut sampling conditions, the number of DEGs was even higher when mapping on the Gmm genome than initially recorded. Many Gmm genes annotated as “Hypothetical” were mapped and annotated on many distinct databases allowing some of them to be properly identified. We identify *Glossina* fly candidate genes encoding (a) a broad panel of proteases as well as (b) chitin—binding proteins, (c) antimicrobial peptide production—Pro3 protein, transferrin, mucin, atttacin, cecropin, etc—to further select in functional studies, the objectives being to probe and validated fly genome manipulation that prevents the onset of the developmental program of one or the other *T. brucei* spp. stumpy form sampled by one of the other bloodfeeding *Glossina* subspecies.

## Introduction

Trypanosomes causing either Human African Trypanosomiasis (HAT, i.e., sleeping sickness) or Animal African Trypanosomiasis (AAT, i.e., Nagana) are transmitted by *Glossina* spp. (tsetse flies). These hematophagous flies acquire their parasite during a blood meal on an infected host, and transmit the mature form of the parasite to another host during a subsequent blood meal. Two forms of HAT have been reported: a chronic and an acute form (Hoare, 1972; Aksoy et al., 2014; Beschin et al., 2014). The chronic form, spread throughout 24 sub-Saharan countries of West Africa, is caused by *Trypanosoma brucei gambiense* (Tbg) and is transmitted by *Glossina palpalis*; this form represents over 90% of all sleeping sickness cases (Welburn et al., 2009). The acute form, endemic to 12 East African countries, is caused by *Trypanosoma brucei rhodesiense* (Tbr), and is transmitted by *Glossina morsitans morsitans* (Gmm). Currently the disease persists in sub-Saharan countries (Louis et al., 2002), where more than 60 million people are exposed to the trypanosomiasis risk. Progress in deciphering the mechanisms of host-parasite interactions involves identifying the genes encoding the factors that govern tsetse fly vector competence (Vickerman et al., 1988; Maudlin and Welburn, 1994; Van den Abbeele et al., 1999), which may promote the development of anti-vector strategies that are alternative or complementary to current strategies.

Using a microarray approach, we recently investigated the effect of trypanosome ingestion by *G. palpalis gambiensis* (Gpg) flies on the transcriptome signatures of *Sodalis glossinidius* (Farikou et al., 2010; Hamidou Soumana et al., 2014a) and *Wigglesworthia glossinidia* (Hamidou Soumana et al., 2014b), two symbionts of tsetse flies (Aksoy et al., 2014). The aim of this previous work was to identify the genes that are differentially expressed in trypanosome infected vs. non-infected or self-cured (refractory) flies and that, consequently, can be suspected to positively or negatively control fly infection. Similarly, using the RNA-seq *de novo* assembly approach, we investigated the differential expression of *G. p. gambiensis* genes in flies challenged or not with trypanosomes (Hamidou Soumana et al., 2015). Furthermore, transcriptome profiling of *T. b. brucei* development in Gmm has recently been reported (Savage et al., 2016).

Since the acute form of HAT is caused by the Gmm/Tbr vector/parasite “couple,” the identification of molecular targets common to both Gpg and Gmm (i.e., orthologous genes) deserves further consideration. Indeed, identification of these targets would allow the development of common approaches to fight both forms of HAT. As Gpg and Gmm are two separate *Glossina* species, their genomes should display some differences between each other. Furthermore, the Gmm genome and the sequences of the Gpg RNA-seq *de novo* assembled genes have been annotated with reference to two distinct database sets: the first set comprises *Drosophila melanogaster, Aedes aegypti, Anopheles gambiae, Culex quinquefasciatus*, and *Phlebotomus papatasi* (International Glossina Genome Initiative, 2014), whereas the second set comprises *Ceratitis capitata, Drosophila melanogaster, D. willistoni, D. virilis, D. mojavensis, Acyrthosiphon pisum, Hydra magnipapillata, Anopheles* sp., *Bombyx* sp., *Aedes* sp., and *Glossina morsitans* (data that were available before the publication of the whole genome sequence; Hamidou Soumana et al., 2015). This indicates that only the *D. melanogaster* database was common to the two database sets used to annotate the differentially expressed Gpg genes and the Gmm genome, respectively. Thus, for the present study, it was necessary to map the sequences of the Gpg RNA-seq *de novo* assembled genes on the Gmm genome and annotate them on the corresponding database. This has been achieved, and the Gpg genes that were previously shown to be differentially expressed (i.e., stimulated vs. non-stimulated flies, and infected vs. non-infected flies; Hamidou Soumana et al., 2015) were annotated on the Gmm database. Finally, the data resulting from the best hits annotation, which provide a translation product for each gene (and thus its potential biological function and physiological role), were compared with data resulting from the previous annotation of the same genes on the set of above-mentioned databases. The overall results provide a data platform that can be applied for further identification of candidate genes involved in the vector competence of both fly species. Importantly, these data could represent promising targets in the development of new anti-vector strategies in the fight against the chronic or acute forms of sleeping sickness.

## Materials and methods

### Ethical statement

All animal experiments in this report were conducted according to internationally recognized guidelines. The experimental protocols were approved by the Ethics Committee on Animal Experiments and the Veterinary Department of the Centre International de Recherche Agronomique pour le Développement (CIRAD; Montpellier, France).

### Sample processing, RNA-Seq library preparation, and sequencing

**Figure 1:**
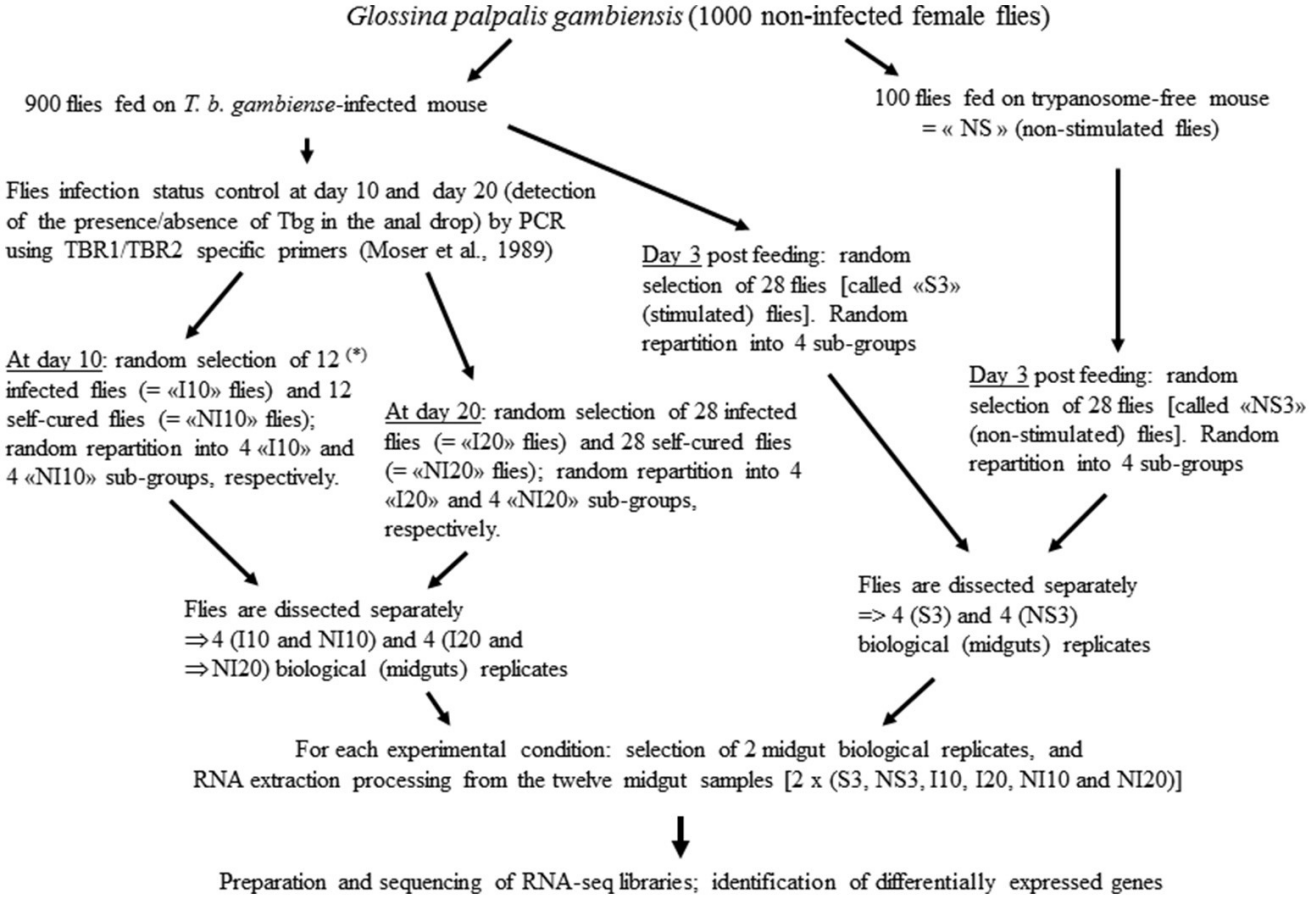
**Samples processing**. (^*^) at day 10 the rate of infected flies was law, thus only 12 infected flies (“I10”) could be sampled (instead of 28 at day 20). Self-cured flies are flies that have ingested trypanosomes (while they have taken a blood meal on an infected mouse), but the anal drops of which were trypanosome negative.

Samples for this study were previously used to identify the differentially expressed genes (DEGs) in Gpg. The different steps are described in the corresponding report (Hamidou Soumana et al., 2015), as well as *pro parte* in reports related to the differential expression of *S. glossinidius* and *W. glossinidia* genes (Hamidou Soumana et al., 2014a,b). Sample processing is summarized in Figure 1.

#### Preparation and sequencing of the RNA-Seq libraries

The sequential steps consisted of: RNA extraction from the pooled midguts of each biological replicate, resuspension of RNA pellets in nuclease-free water, concentration, RNA quantification, and quality control (to confirm the absence of any DNA contamination).

#### Generation of RNA-Seq libraries

RNA-seq libraries were generated using the Illumina TruSeq™ RNA Sample Preparation Kit (Illumina; San Diego, USA). The sequential steps consisted of: mRNA purification from 4 μg total RNA using poly-T oligo-linked magnetic beads; fragmentation of RNA using divalent cations under elevated temperature (Illumina fragmentation buffer); first-strand cDNA synthesis using random oligonucleotides and SuperScript II; second-strand cDNA synthesis using DNA Polymerase I and RNase H; conversion of remaining overhangs into blunt ends via exonuclease/polymerase activities and enzyme removal; and adenylation of 3′ ends of cDNA fragments, with ligation of Illumina PE adapter oligonucleotides for further hybridization. Finally, cDNA fragments were selected (preferably 200 bp in length) in which fragments with ligated adaptor molecules on both ends were selectively enriched using Illumina PCR Primer Cocktail, and the products were purified and quantified using the Agilent DNA assay on the Agilent Bioanalyzer 2100 system.

#### Brief summary of the pipeline for generating quality-controlled reads

A total of 12 RNA-seq libraries were prepared, sequenced, and compared, including two biological replicates for each of the NS3, S3, I10, NI10, I20, and I20 samples. Clustering of the index-coded samples was performed on a cBot Cluster Generation System using TruSeq PE Cluster Kit-cBot-HS (Illumina). After cluster generation, the library preparations were sequenced on an Illumina Hiseq 2000 platform, and 100-bp paired-end reads were generated. Image analyses and base calling were performed using the Illumina HiSeq Control Software and Real-Time Analysis component. Demultiplexing was performed using CASAVA 1.8.2. The quality of the raw data was assessed using FastQC (Babraham Institute) and the Illumina software SAV (Sequencing Analysis Viewer). Raw sequencing reads from this study were exported in the FASTQ format and were deposited at the NCBI Short Read Archive (SRA) with the accession number SRP046074; aligned BAM files are available on request.

### Identification of DEGs once the reads generated from the 12 Gpg fly gut RNA seq libraries were mapped and annotated on a panel of non-insect and insect genome databases, one of them being Gmm

The RNA-seq reads that satisfied the quality control (i.e., removal of ambiguous nucleotides, low-quality sequences with quality scores <20, and sequences <15 bp in length) were mapped on the *G. m. morsitans* genome (13,807 scaffolds; International Glossina Genome Initiative, 2014) from VectorBase (www.vectorbase.org) and GenBank (accession no. CCAG010000000). This was achieved via the splice junction mapper TopHat 2.0.13 (Kim et al., 2013) using Bowtie 2.1.0 (Langmead and Salzberg, 2012), to align RNA-seq reads to the *Glossina morsitans* genome (GmorY1 assembly, release date: January 2014). Final read alignments with more than 12 mismatches were discarded.

Gene counting (number of reads aligned on each gene) was performed before statistical analysis, using HTSeq count 0.5.3p9 (union mode; Anders et al., 2014). Genes with <10 reads (cumulating all analyzed samples) were filtered and removed. We used the Bioconductor (Gentleman et al., 2004) software package EdgeR (Robinson et al., 2010) 3.6.7. to identify genes displaying a modified expression profile as a result of fly infection by trypanosomes. Data were normalized using the upper quartile normalization factors, using the quartiles method (Bullard et al., 2010). Genes with an adjusted *p* < 5% according to the False Discovery Rate (FDR) method from Benjamini and Hochberg (1995) were declared differentially expressed.

### Bio informatics-based approaches aimed to identify molecular DEGs in both Gmm and Gpg once the latter are subverted as T. brucei spp hosts *per se*

Tsetse fly gene orthologs were tentatively identified using BLAST searches (Mount, 2007) with annotation against the NCBI non-redundant (Nr) sequence database, using an *E*-value cut-off of 10^−5^ (*E* < 0.00001), according to the best hits against known sequences. This was performed to retrieve orthologous genes with the highest sequence similarity to the given unigenes along with putative functional annotations. The official gene symbols of tsetse fly gene orthologs were used for functional annotation. Along with Nr annotations, the “Database for Annotation, Visualization and Integrated Discovery” (DAVID; Dennis et al., 2003) was used to obtain GO annotations of unigenes. The KEGG pathway annotations of tsetse fly gene orthologs were performed using the BLASTX software against the KEGG database (Wixon and Kell, 2000).

Analyzing the two annotation processes of the Gpg DEGs consisted in comparing the list of the “best hits” resulting from the Gpg DEG annotation on the Gmm database with the list resulting from the Gpg DEG annotation previously performed on a set of other databases (*Ceratitis capitata, Drosophila melanogaster, D. willistoni, D. virilis, D. mojavensis, Acyrthosiphon pisum, Hydra magnipapillata, Anopheles* sp., *Bombyx* sp., *Aedes* sp., and *Glossina morsitans*; Hamidou Soumana et al., 2015). The first step consisted in mixing the DEGs identified at the three experimental times (3, 10, and 20 days) and removing the duplicates, so as to take into account all recorded DEGs except for one of each. The second step consisted in removing the DEGs in which the annotation (best hit) resulted in “hypothetical” or “uncharacterized” proteins, as well as those identified with a numerical identifier, in order to only consider identified and named proteins. Finally, the names of the proteins (best hits) were standardized and alphabetically classified. This process was performed separately for the DEGs annotated with reference to the Gmm database, as well as those previously annotated on the above-characterized set of other databases. The two final listings were then combined (Microsoft Excel software), and their content was arranged according to the alphabetical order of protein names. This procedure facilitated the detection of the best hits that are common to both annotation processes and their corresponding genes.

## Results

### Mapping of PolyA+ mRNA

A total of 459,555,846 clusters were generated from the 12 RNA-seq libraries. Quality controls were performed to ensure the reliability of the libraries after removal of ambiguous nucleotides, low-quality sequences (quality scores < 20), and sequences <15 bp in length. Finally, 436,979,101 clean clusters were obtained (Table 1). Clean reads had Phred-like quality scores at the Q20 level (i.e., a sequencing error probability of 0.01). These clean sequenced reads with no strand-specificity were mapped to the Gmm reference genome using TopHat (with Bowtie 2) software in order to identify exon-exon splice junctions and to ensure enough sensitivity in mapping reads with polymorphisms.

**Table 1:** **Assembly quality of Gpg libraries at the three different sampling times**.

**Samples**	**Number of crude clusters (CC)**	**Number of clusters after filtering (CAF)**	**% CAF/CC**
NS 3-day sample^a^	36,002,596	34,386,734	95.51
NS 3-day sample^b^	41,153,580	39,330,015	95.57
S 3-day sample	32,726,727	31,257,269	95.51
S 3-day sample	33,386,646	31,848,385	95.39
NI 10-day sample	33,159,650	31,593,962	95.28
NI 10-day sample	30,632,671	29,185,036	95.27
I 10-day sample	42,223,049	40,108,756	94.99
I 10-day sample	43,418,918	41,279,341	95.07
NI 20-day sample	41,882,170	39,688,764	94.76
NI 20-day sample	38,192,692	36,205,087	94.80
I 20-day sample	40,587,354	38,401,915	94.62
I 20-day sample	46,189,793	43,693,837	94.60
Total	459,555,846	436,979,101	–
Mean	38,296,320	36,414,925	95.08

*The superscipts ^a^ and ^b^ are two replicates of the “non-stimulated samples” at day 3. Idem for the other sampling conditions. S, stimulated; NS, non-stimulated; NI, non-infected; I, infected*.

**Figure 2:**
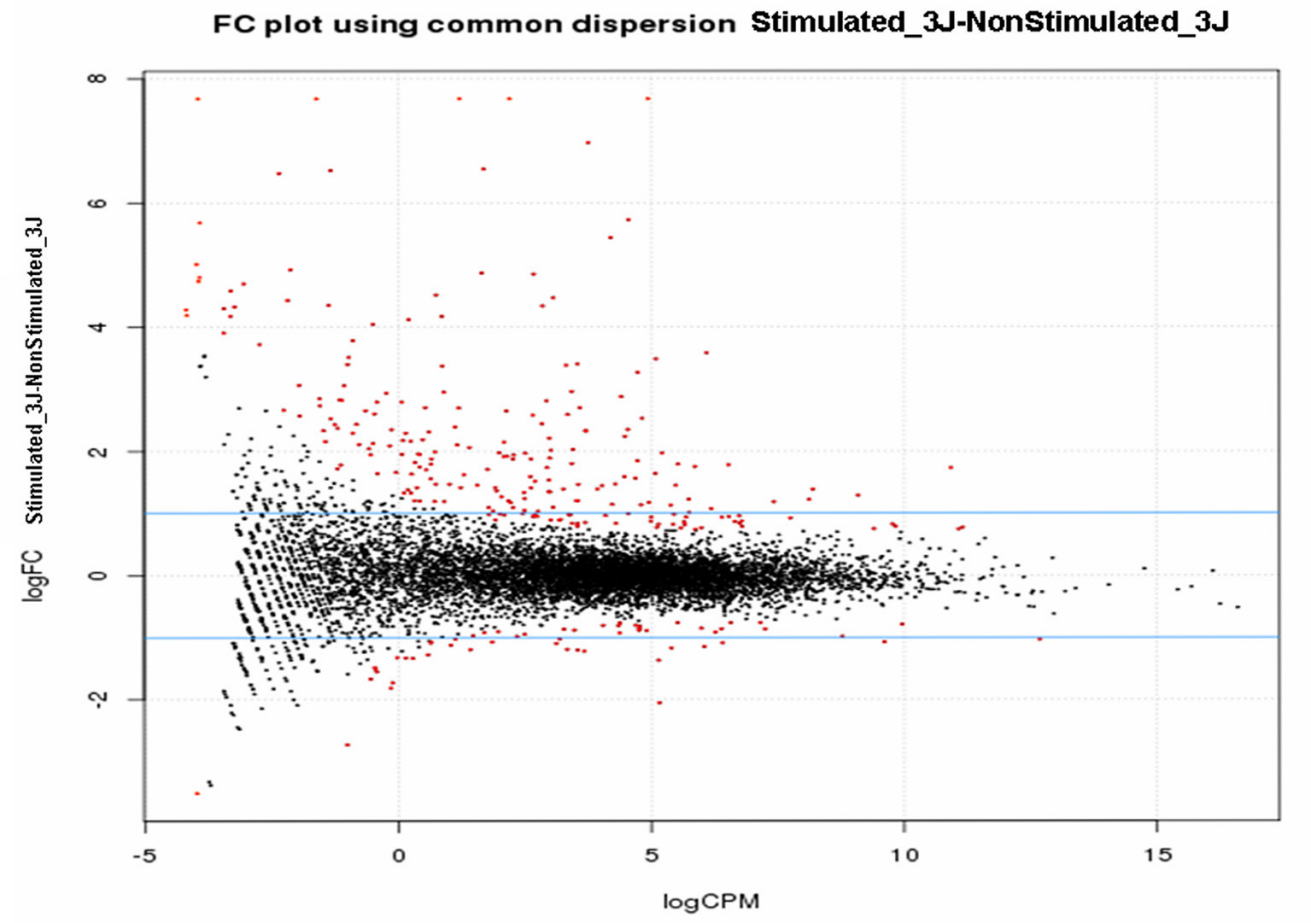
**Smear plot of libraries**. Samples are from trypanosome stimulated-/-non-stimulated tsetse fly midguts sampled 3 days post-infected blood meal, after normalization using the upper quartile method.

Filtering and removing any genes with <10 mapped reads allowed mapping 8,286 (stimulated vs. non-stimulated flies; 3 days), 8,032 (infected vs. refractory flies; 10 days) and 8,101 Gpg genes (infected vs. refractory flies; 20 days) on the Gmm reference genome (International Glossina Genome Initiative, 2014). Further, analyses to reveal differential expression (DE) were performed using the bioinformatics tools HTseq and EdgeR from Bioconductor (http://www.bioconductor.org/), which use the R statistical programming language and are widely accepted for modeling the inherent variation between biological replicates. Figure 2 presents the log_2_ fold-change (stimulated vs. non-stimulated flies at day-3 post-infected blood meal) against the log_2_ of the reads concentration (log-counts-per-million) for each gene after normalization. The generated cloud shows a log fold-change centered on 0 (ordinate axis), signifying that the libraries are properly normalized. Genes that are differentially expressed between the S and NS samples (*p* < 0.05) are represented in red. Similar results were obtained for the other experimental conditions.

### Identification of DEGs and functional annotation

The EdgeR method identified a total of 284, 139, and 59 Gmm genes corresponding respectively to the Gpg DEG samples S3 vs. NS3 (Supplementary Table S1), I10 vs. NI10 (Supplementary Table S2), and I20 vs. NI20 (Supplementary Table S3), at a *p* < 0.05. Most of these genes were overexpressed regardless of the experimental condition. Specifically, there were 229 out of 284 genes (80.6%) in the day-3 samples (S3 vs. NS3), 119 out of 139 genes (85.6%) in I-10 vs. NI-10 samples, and 37 out of 59 genes (62.7%) in I20 vs. NI20. Furthermore, the number of DEGs were highly differentially overexpressed (log_2_ FC > 2) or underexpressed (log_2_ FC < –2). Specifically, there were 97 out of 284 DEGs (34%; S3 vs. NS3), 60 out of 139 DEGs (43%; I10 vs. NI10), and 19 out of 59 DEGs (32%; I20 vs. NI20). These data are summarized in Table 2. Genes exhibiting a highly differential overexpression or underexpression under the different experimental conditions (i.e., S vs. NS, I10 vs. NI10, and I20 vs. NI20) are grouped together in Table 3. Most DEGs encode a wide range of proteases, although 91 DEGs presented in Supplementary Tables S1–S3 could not be properly annotated (i.e., best hit description = “hypothetical”), signifying that the panel of databases used for the annotation process should be enlarged or that the genes may be specific to the Gmm genome. In addition, several of the DEGs were very highly overexpressed. For example the log_2_ FC of GMOY009756, which encodes a trypsin, had a fold-change of 7.14 in S3 vs. NS3 samples, and GMOY002278, which encodes the proteinase inhibitor I2, had a fold-change of 9.47 in I10 vs. NI10. In contrast, some DEGs were underexpressed: the log_2_ FC of GMOY005345, which encodes an aspartic peptidase, had a fold-change of –6.51 in I20 vs. NI20 samples. Table 3 is presented so as to facilitate comparison of differential expression levels for a given gene along the three sampling times. For instance, the levels (in log_2_ FC) of GMOY005345, which encodes an aspartic peptidase, are 3.39 (S3 vs. NS3), 2.70 (I10 vs. NI10), and –6.51 (I20 vs. NI20).

**Table 2:** **Number of differentially expressed genes in Gpg**.

**Experimental conditions**	**Number of identified genes**	**Significantly differentially expressed genes**			
		**Overall**	**Overexpressed**	**Fold-change**	
				**2 < log_2_ FC or log_2_ FC < –2**	**3 < log_2_ FC or log_2_ FC < –3**
S vs. NS (3 days)	8,286	284	229 (80.6%)	97 (34.1%)	44 (15.5%)
I vs. NI (10 days)	8,032	139	119 (85.6%)	60 (43.1%)	35 (25.2%)
I vs. NI (20 days)	8,101	59	37 (62.7%)	19 (32.2%)	6 (10.2%)

*S, stimulated; NS, non-stimulated; NI, non-infected; I: infected*.

**Table 3:** **Annotation on the ***Glossina morsitans morsitans*** genome of Gpg genes differentially expressed in response to Tbg infection**.

**Genes**	**Fold-change log_2_ FC**	**Encoded proteins (best hits)**	**Gene Ontology (GO)**		
			**Biological process**	**Molecular function**	**Cellular component**
**PROTEASES AND PROTEASE INHIBITORS**					
GMOY005345	3.39	Aspartic peptidase	GO:0006508 proteolysis	GO:0004190 aspartic-type endopeptidase activity	No terms assigned
GMOY005345	2.70	Aspartic peptidase	GO:0006508 proteolysis	GO:0004190 aspartic-type endopeptidase activity	No terms assigned
GMOY005345	–6.51	Aspartic peptidase	GO:0006508 proteolysis	GO:0004190 aspartic-type endopeptidase activity	No terms assigned
GMOY007305	2.09	Destabilase	No terms assigned	GO:0003796 lysozyme activity	No terms assigned
GMOY007305	3.00	Destabilase	No terms assigned	GO:0003796 lysozyme activity	No terms assigned
GMOY000103	2.64	Fat body c-type lysozyme	No terms assigned	No terms assigned	No terms assigned
GMOY000103	2.74	Fat body c-type lysozyme	No terms assigned	No terms assigned	No terms assigned
GMOY002036	–2.75	Peptidase S1A, chymotrypsin-type	GO:0006508 proteolysis	GO:0004252 serine-type endopeptidase activity	No terms assigned
GMOY003273	2.30	Peptidase S1A, chymotrypsin-type	GO:0006508 proteolysis	GO:0004252 serine-type endopeptidase activity	No terms assigned
GMOY003994	4.19	Peptidase S1A, chymotrypsin-type	GO:0006508 proteolysis	GO:0004252 serine-type endopeptidase activity	No terms assigned
GMOY006266	3.72	Peptidase S1A, chymotrypsin-type	GO:0006508 proteolysis	GO:0004252 serine-type endopeptidase activity	No terms assigned
GMOY008964	3.32	Peptidase S1A, chymotrypsin-type	GO:0006508 proteolysis	GO:0004252 serine-type endopeptidase activity	No terms assigned
GMOY008965	3.52	Peptidase S1A, chymotrypsin-type	GO:0006508 proteolysis	GO:0004252 serine-type endopeptidase activity	No terms assigned
GMOY008966	3.35	Peptidase S1A, chymotrypsin-type	GO:0006508 proteolysis	GO:0004252 serine-type endopeptidase activity	No terms assigned
GMOY008966	4.41	Peptidase S1A, chymotrypsin-type	GO:0006508 proteolysis	GO:0004252 serine-type endopeptidase activity	No terms assigned
GMOY009436	2.11	Peptidase S1A, chymotrypsin-type	GO:0006508 proteolysis	GO:0004252 serine-type endopeptidase activity	No terms assigned
GMOY009757	2.76	Peptidase S1A, chymotrypsin-type	GO:0006508 proteolysis	GO:0004252 serine-type endopeptidase activity	No terms assigned
GMOY010768	2.73	Peptidase S1A, chymotrypsin-type	GO:0006508 proteolysis	GO:0004252 serine-type endopeptidase activity	No terms assigned
GMOY010768	2.06	Peptidase S1	GO:0006508 proteolysis	GO:0004252 serine-type endopeptidase activity	No terms assigned
GMOY002729	3.01	Serine protease 1	GO:0006508 proteolysis	GO:0004252 serine-type endopeptidase activity	No terms assigned
GMOY000672	6.95	Serine protease 6	GO:0006508 proteolysis	GO:0004252 serine-type endopeptidase activity	No terms assigned
GMOY000672	6.78	Serine protease 6	GO:0006508 proteolysis	GO:0004252 serine-type endopeptidase activity	No terms assigned
GMOY009756	7.14	Trypsin	GO:0006508 proteolysis	GO:0004252 serine-type endopeptidase activity	No terms assigned
GMOY009756	3.48	Trypsin	GO:0006508 proteolysis	GO:0004252 serine-type endopeptidase activity	No terms assigned
GMOY008967	2.55	Trypsin	GO:0006508 proteolysis	GO:0004252 serine-type endopeptidase activity	No terms assigned
GMOY008967	3.37	Trypsin-like cysteine/serine peptid. domain	No terms assigned	GO:0003824 catalytic activity	No terms assigned
GMOY010488	6.83	Imune reactive putative protease inhibitor	No terms assigned	No terms assigned	No terms assigned
GMOY010488	4.69	Immune reactive putative protease inhibitor	No terms assigned	No terms assigned	No terms assigned
GMOY002277	2.31	Proteinase inhibitor I2, Kunitz metazoa	No terms assigned	GO:0004867 serine-type endopeptidase inhibit. Activ.	No terms assigned
GMOY002277	4.10	Proteinase inhibitor I2, Kunitz metazoa	No terms assigned	GO:0004867 serine-type endopeptidase inhibit. Activ.	No terms assigned
GMOY002278	6.54	Proteinase inhibitor I2, Kunitz metazoa	No terms assigned	GO:0004867 serine-type endopeptidase inhibit. Activ.	No terms assigned
GMOY002278	9.47	Proteinase inhibitor I2, Kunitz metazoa	No terms assigned	GO:0004867 serine-type endopeptidase inhibit. Activ.	No terms assigned
GMOY008344	2.94	Trypsin Inhibitor-like	No terms assigned	No terms assigned	No terms assigned
GMOY008344	4.38	Trypsin Inhibitor-like	No terms assigned	No terms assigned	No terms assigned
**ESTERASES—HYDROLASES**					
GMOY000067	3.35	Alkaline phosphatase	GO:0008152 metabolic process	GO:0016791 phosphatase activity	No terms assigned
GMOY000067	3.60	Alkaline phosphatase	GO:0008152 metabolic process	GO:0003824 catalytic activity	No terms assigned
GMOY004731	2.06	Alkaline phosphatase-like, alpha/beta/alpha	GO:0008152 metabolic process	GO:0003824 catalytic activity	No terms assigned
GMOY006875	–2.02	Alkaline phosphatase-like, alpha/beta/alpha	GO:0008152 metabolic process	GO:0003824 catalytic activity	No terms assigned
GMOY004236	2.36	Acylphosphatase-like	No terms assigned	GO:0003998 acylphosphatase activity	No terms assigned
GMOY006958	2.60	Carboxylesterase	No terms assigned	No terms assigned	No terms assigned
GMOY011249	2.83	Carboxylesterase	No terms assigned	No terms assigned	No terms assigned
GMOY012368	2.53	Exonuclease	No terms assigned	No terms assigned	No terms assigned
GMOY007402	3.85	Extracellular Endonuclease, subunit A	No terms assigned	GO:0016787 hydrolase activity	No terms assigned
GMOY012360	–2.93	Extracellular Endonuclease, subunit A	No terms assigned	GO:0016787 hydrolase activity	No terms assigned
GMOY009375	7.56	Glycoside hydrolase	GO:0005975 carbohyd. metabolic process	GO:0003824 catalytic activity	No terms assigned
GMOY012361	–2.55	Tsal2 protein precursor	No terms assigned	GO:0016787 hydrolase activity	No terms assigned
GMOY004309	2.44	Thiolase-like	GO:0008152 metabolic process	GO:0003824 catalytic activity	No terms assigned
GMOY007148	2.10	Thiolase-like	GO:0008152 metabolic process	GO:0003824 catalytic activity	No terms assigned
**BINDING**					
GMOY010194	4.12	Araucan	No terms assigned	GO:0003677 DNA binding	No terms assigned
GMOY009525	7.53	Armadillo-type fold	No terms assigned	GO:0005488 binding	No terms assigned
GMOY009611	2.60	Barrier- to-autointegration factor, BAF	No terms assigned	GO:0003677 DNA binding	No terms assigned
GMOY009394	–5.56	Basic-leucine zipper domain	GO:0006355 regulation of transcription	GO:0003700 sequence-specific	No terms assigned
GMOY010195	5.99	Caupolican	GO:0006355 regul. of transcrip,DNA-templated	GO:0003677 DNA binding	GO:0005634 nucleus
GMOY002708	7.89	Chitin binding	GO:0006030 chitin metabolic process	GO:0008061 chitin binding	GO:0005576 extracel
GMOY005278	2.93	Chitin binding domain	GO:0006030 chitin metabolic process	GO:0008061 chitin binding	GO:0005576 extracel
GMOY003840	4.12	Chitin binding domain	GO:0006030 chitin metabolic process	GO:0008061 chitin binding	GO:0005576 extracel
GMOY011054	6.08	Chitin binding domain	GO:0006030 chitin metabolic process	GO:0008061 chitin binding	GO:0005576 extracel
GMOY011810	6.55	Chitin binding domain	GO:0006030 chitin metabolic process	GO:0008061 chitin binding	GO:0005576 extracel
GMOY011809	8.08	Pro1 (Chitin related)	GO:0006030 chitin metabolic process	GO:0008061 chitin binding	GO:0005576 extracel
GMOY004647	4.24	Cupredoxin	No terms assigned	GO:0005507 copper ion binding	No terms assigned
GMOY004364	2.90	Haemolymph juvenile hormone binding	No terms assigned	No terms assigned	No terms assigned
GMOY005487	3.48	Lim3	No terms assigned	GO:0008270 zinc ion binding	No terms assigned
GMOY007084	2.39	NAD(P)-binding domain	No terms assigned	No terms assigned	No terms assigned
GMOY002356	2.31	Nucleotide-binding	No terms assigned	GO:0000166 nucleotide binding	No terms assigned
GMOY002825	4.47	Odorant binding protein 2	No terms assigned	GO:0005549 odorant binding	No terms assigned
GMOY002825	2.03	Odorant binding protein 2	No terms assigned	No terms assigned	No terms assigned
GMOY005548	2.99	Odorant binding protein 7	No terms assigned	No terms assigned	No terms assigned
GMOY001476	2.20	Odorant binding protein 22	No terms assigned	GO:0005549 odorant binding	No terms assigned
GMOY008769	4.95	Small GTPase	GO:0007165 signal transduction	GO:0005525 GTP binding	GO:0016020 membrane
GMOY004228	5.44	Transferrin family	GO:0006879 cellular iron ion homeostasis	GO:0008199 ferric iron binding	GO:0005576 extracel
GMOY004228	2.63	Transferrin family, iron binding site	GO:0006879 cellular iron ion homeostasis	GO:0008199 ferric iron binding	GO:0005576 extracel
GMOY008315	2.05	Winged helix-turn-helix DNA-binding domain	GO:0006355 regul. of transcrip,DNA-templated	GO:0043565 sequence-specific DNA binding	No terms assigned
				Transcription Factor Activity	
**TRANSPORT/TRANSFERASE ACTIVITY**					
GMOY004684	–2.39	Cellul. retinaldehyde binding/a-tocopherol transport	GO:0006810 transport	GO:0005215 transporter activity	GO:0005622 intracel
GMOY008601	2.58	Fatty acid synthase 3	GO:0008152 metabolic process	GO:0016740 transferase activity	No terms assigned
GMOY008601	4.11	Fatty acid synthase 3	GO:0008152 metabolic process	GO:0016740 transferase activity	No terms assigned
GMOY008602	2.02	Fatty acid synthase 4	GO:0008152 metabolic process	GO:0016740 transferase activity	No terms assigned
GMOY008602	–2.55	Fatty acid synthase 4	GO:0008152 metabolic process	GO:0016740 transferase activity	No terms assigned
GMOY005442	2.35	Lipid transport protein	GO:0006869 lipid transport	GO:0005319 lipid transporter activity	No terms assigned
GMOY005442	2.40	Lipid transport protein	GO:0006869 lipid transport	GO:0005319 lipid transporter activity	No terms assigned
GMOY003490	4.50	Major Facilitator Superfamily transporter	GO:0055085 transmembrane transport	No terms assigned	GO:0016021 integral
GMOY003491	3.97	Major Facilitator Superfamily transporter	GO:0055085 transmembrane transport	No terms assigned	GO:0016021 integral
GMOY005103	2.77	Major Facilitator Superfamily transporter	GO:0055085 transmembrane transport	No terms assigned	GO:0016021 integral
GMOY007627	2.09	Major Facilitator Superfamily transporter	GO:0055085 transmembrane transport	No terms assigned	GO:0016021 integral
GMOY005102	6.28	N-acetylgalactosaminyltransferase	GO:0008152 metabolic process	No terms assigned	No terms assigned
GMOY011877	2.37	Na+ channel, amiloride-sensitive	GO:0006814 sodium ion transport	GO:0005272 sodium channel activity	GO:0016020 membrane
GMOY009903	2.75	Neurotransmitter-gated ion-channel	GO:0006811 ion transport	No terms assigned	GO:0016021 integral
GMOY005934	2.72	Pyridoxal phosphate-dependent transferase	No terms assigned	GO:0003824 catalytic activity	No terms assigned
GMOY009343	8.14	Sodium:neurotransmitter symporter	GO:0006836 neurotransmitter transport	GO:0005328 neurotransmitter:Na symporter act	GO:0016021 integral
GMOY009343	6.43	Sodium:neurotransmitter symporter	GO:0006836 neurotransmitter transport	GO:0005328 neurotransmitter:Na symporter act	GO:0016021 integral
GMOY009386	2.44	Sodium:neurotransmitter symporter	GO:0006836 neurotransmitter transport	GO:0005328 neurotransmitter:Na symporter act	GO:0016021 integral
GMOY002486	2.59	Two pore domain K channel, TASK family	GO:0071805 K ion transmemb, transport	GO:0005267 potassium channel activity	GO:0016020 membrane
GMOY012088	4.05	Tyrosine aminotransferase	GO:0009072 aromatic amino acid	GO:0004838 L-tyrosine:2-oxoglutarate	No terms assigned
			family metabolic process	aminotransferase Activity	
**OXIDO-REDUCTION PROCESS**					
GMOY001939	2.50	Cytochrome P450-4g1	GO:0055114 oxidation-reduction process	GO:0016705 oxidoreductase activity	No terms assigned
GMOY002598	2.15	Cytochrome P450	GO:0055114 oxidation-reduction process	GO:0016705 oxidoreductase activity	No terms assigned
GMOY006475	2.28	Cytochrome P450-4g1	GO:0055114 oxidation-reduction process	GO:0016705 oxidoreductase activity	No terms assigned
GMOY006761	2.42	Cytochrome P450-4g1	GO:0055114 oxidation-reduction process	GO:0016705 oxidoreductase activity	No terms assigned
GMOY006761	–2.08	Cytochrome P450-4g1	GO:0055114 oxidation-reduction process	GO:0016705 oxidoreductase activity	No terms assigned
GMOY007181	3.49	Cytochrome P450	GO:0055114 oxidation-reduction process	GO:0016705 oxidoreductase activity	No terms assigned
GMOY007652	3.43	Cytochrome P450	GO:0055114 oxidation-reduction process	GO:0016705 oxidoreductase activity	No terms assigned
GMOY009767	3.96	Cytochrome P450	GO:0055114 oxidation-reduction process	GO:0016705 oxidoreductase activity	No terms assigned
GMOY009909	3.35	Cytochrome P450	GO:0055114 oxidation-reduction process	GO:0016705 oxidoreductase activity	No terms assigned
GMOY007529	4.49	Dehydrogenase/reductase	GO:0008152 metabolic process	GO:0016491 oxidoreductase activity	No terms assigned
GMOY004332	2.12	Fatty acyl-CoA reductase	No terms assigned	GO:0080019 fatty-acyl-CoA reductase activity	No terms assigned
GMOY007497	6.25	NADH-cytochrome b-5 reductase 2	GO:0055114 oxidation-reduction process	GO:0016491 oxidoreductase activity	No terms assigned
GMOY010446	2.40	2-oxoglutarate dioxygenase	GO:0055114 oxidation-reduction process	GO:0050353 trimethyllysine dioxygenase activity	No terms assigned
**HYPOTHETICAL**					
GMOY000215	4.17	Hypothetical	No terms assigned	No terms assigned	No terms assigned
GMOY000215	5.24	Hypothetical	No terms assigned	No terms assigned	No terms assigned
GMOY000257	2.90	Hypothetical	No terms assigned	No terms assigned	No terms assigned
GMOY001239	2.53	Hypothetical	No terms assigned	No terms assigned	No terms assigned
GMOY002434	3.22	Hypothetical	No terms assigned	No terms assigned	No terms assigned
GMOY002933	2.07	Hypothetical	No terms assigned	No terms assigned	No terms assigned
GMOY002986	2.69	Hypothetical	No terms assigned	No terms assigned	No terms assigned
GMOY003011	2.30	Hypothetical	No terms assigned	No terms assigned	No terms assigned
GMOY003030	4.38	Hypothetical	No terms assigned	No terms assigned	No terms assigned
GMOY003034	–2.10	Hypothetical	No terms assigned	No terms assigned	No terms assigned
GMOY003158	2.70	Hypothetical	No terms assigned	No terms assigned	No terms assigned
GMOY003197	2.04	Hypothetical	No terms assigned	No terms assigned	No terms assigned
GMOY003830	2.75	Hypothetical	No terms assigned	No terms assigned	No terms assigned
GMOY003830	3.68	Hypothetical	No terms assigned	No terms assigned	No terms assigned
GMOY003974	2.28	Hypothetical	No terms assigned	No terms assigned	No terms assigned
GMOY003976	3.89	Hypothetical	No terms assigned	No terms assigned	No terms assigned
GMOY004022	–2.06	Hypothetical	No terms assigned	No terms assigned	No terms assigned
GMOY004337	5.80	Hypothetical	No terms assigned	No terms assigned	No terms assigned
GMOY004337	6.61	Hypothetical	No terms assigned	No terms assigned	No terms assigned
GMOY005055	6.08	Hypothetical	No terms assigned	No terms assigned	No terms assigned
GMOY005606	6.32	Hypothetical	No terms assigned	No terms assigned	No terms assigned
GMOY005797	6.60	Hypothetical	No terms assigned	No terms assigned	No terms assigned
GMOY005797	6.49	Hypothetical	No terms assigned	No terms assigned	No terms assigned
GMOY005798	3.98	Hypothetical	No terms assigned	No terms assigned	No terms assigned
GMOY005798	6.25	Hypothetical	No terms assigned	No terms assigned	No terms assigned
GMOY005799	2.24	Hypothetical	No terms assigned	No terms assigned	No terms assigned
GMOY006671	4.00	Hypothetical	No terms assigned	No terms assigned	No terms assigned
GMOY006276	2.33	Hypothetical	No terms assigned	No terms assigned	No terms assigned
GMOY007187	3.59	Hypothetical	No terms assigned	No terms assigned	No terms assigned
GMOY007637	4.06	Hypothetical	No terms assigned	No terms assigned	No terms assigned
GMOY008016	4.65	Hypothetical	No terms assigned	No terms assigned	No terms assigned
GMOY008016	6.67	Hypothetical	No terms assigned	No terms assigned	No terms assigned
GMOY008627	3.64	Hypothetical	No terms assigned	No terms assigned	No terms assigned
GMOY009539	2.28	Hypothetical	No terms assigned	No terms assigned	No terms assigned
GMOY009540	2.19	Hypothetical	No terms assigned	No terms assigned	No terms assigned
GMOY009541	2.40	Hypothetical	No terms assigned	No terms assigned	No terms assigned
GMOY009951	3.11	Hypothetical	No terms assigned	No terms assigned	No terms assigned
GMOY010224	6.87	Hypothetical	No terms assigned	No terms assigned	No terms assigned
GMOY010224	3.57	Hypothetical	No terms assigned	No terms assigned	No terms assigned
GMOY010232	–2.44	Hypothetical	No terms assigned	No terms assigned	No terms assigned
xGMOY012069	9.07	Hypothetical	No terms assigned	No terms assigned	No terms assigned
GMOY012069	5.33	Hypothetical	No terms assigned	No terms assigned	No terms assigned
GMOY008956	2.90	hypothetical conserved protein	No terms assigned	No terms assigned	No terms assigned
**MISCELLANEOUS**					
GMOY008458	5.74	Actin-related protein	No terms assigned	No terms assigned	No terms assigned
GMOY008368	2.22	Adipokinetic hormone recept isoform A	No terms assigned	No terms assigned	No terms assigned
GMOY004147	4.32	Apolipophorin-III superfamily	No terms assigned	No terms assigned	No terms assigned
GMOY011562	2.29	Cecropin	No terms assigned	No terms assigned	GO:0005576 extracel
GMOY011562	2.36	Cecropin	No terms assigned	No terms assigned	GO:0005576 extracel
GMOY011563	2.77	Cecropin	No terms assigned	No terms assigned	GO:0005576 extracel
GMOY010882	3.02	Chemosensory protein 3	No terms assigned	No terms assigned	No terms assigned
GMOY007457	2.47	Cytochrome b561/ferric reduct transmembrane	No terms assigned	No terms assigned	GO:0016021 integral
GMOY003354	2.48	Elongase 9	No terms assigned	No terms assigned	GO:0016021 integral
GMOY003354	6.25	Elongase 9	No terms assigned	No terms assigned	GO:0016021 integral
GMOY008821	3.10	Elongase 4	No terms assigned	No terms assigned	GO:0016021 integral
GMOY009277	3.72	Insect cuticle protein	No terms assigned	GO:0042302 structural constituent of cuticle	No terms assigned
GMOY003876	2.08	Insect cuticle protein	No terms assigned	GO:0042302 structural constituent of cuticle	No terms assigned
GMOY011216	2.40	Insect cuticle protein	No terms assigned	GO:0042302 structural constituent of cuticle	No terms assigned
GMOY002258	2.03	Insulin-like	No terms assigned	GO:0005179 hormone activity	GO:0005576 extracel
GMOY003944	9.22	LIM and senesc, cell antigen-like-protein 1	GO:0009987 cellular process	GO:0005198 structural molecule activity	GO:0043226 organelle
GMOY011997	3.02	Mammalian NeuroPept, Y like receptor	No terms assigned	No terms assigned	GO:0016021 integral
GMOY012052	3.40	Mammalian NeuroPept, Y like receptor	No terms assigned	No terms assigned	GO:0016021 integral
GMOY009745	2.02	Milk gland protein 1	No terms assigned	No terms assigned	No terms assigned
GMOY001342	–2.40	Milk gland protein 2	No terms assigned	No terms assigned	No terms assigned
GMOY001342	2.14	Milk gland protein 2	No terms assigned	No terms assigned	No terms assigned
GMOY012125	3.57	Milk gland protein 3	No terms assigned	No terms assigned	No terms assigned
GMOY001343	2.50	Milk gland protein 6	No terms assigned	No terms assigned	No terms assigned
GMOY012016	–4.00	Milk gland protein 8	No terms assigned	No terms assigned	No terms assigned
GMOY012016	2.36	Milk gland protein 8	No terms assigned	No terms assigned	No terms assigned
GMOY012369	2.24	Milk gland protein 10	No terms assigned	No terms assigned	No terms assigned
GMOY010160	2.78	Mpv17/PMP22	No terms assigned	No terms assigned	GO:0016021 integral membrane
GMOY009494	–3.95	Rhodanese-like domain	No terms assigned	No terms assigned	No terms assigned
GMOY010675	2.12	Single domain Von Willebrand factor type C	No terms assigned	No terms assigned	No terms assigned
GMOY007078	2.52	Single domain Von Willebrand factor type C	No terms assigned	No terms assigned	No terms assigned

*Gpg genes that were previously shown to be differentially expressed (DEGs) 3, 10, and 20 days after being challenged with Tbg were mapped on the Gmm genome and annotated on this reference genome. The table presents Gmm genes that are heterologs of the Gpg DEGs, in addition to the annotation results and the gene ontology. Only highly differential expressed genes (log_2_FC < –2 or log_2_FC > 2) have been considered. **Black** fonts: genes that are differentially expressed in stimulated vs. non-stimulated flies (at day 3 after fly challenge). 3952a4**Blue** and ee1f25**Red** fonts: genes differentially expressed at day 10 and day 20 after fly challenge, respectively*.

**Figure 3:**
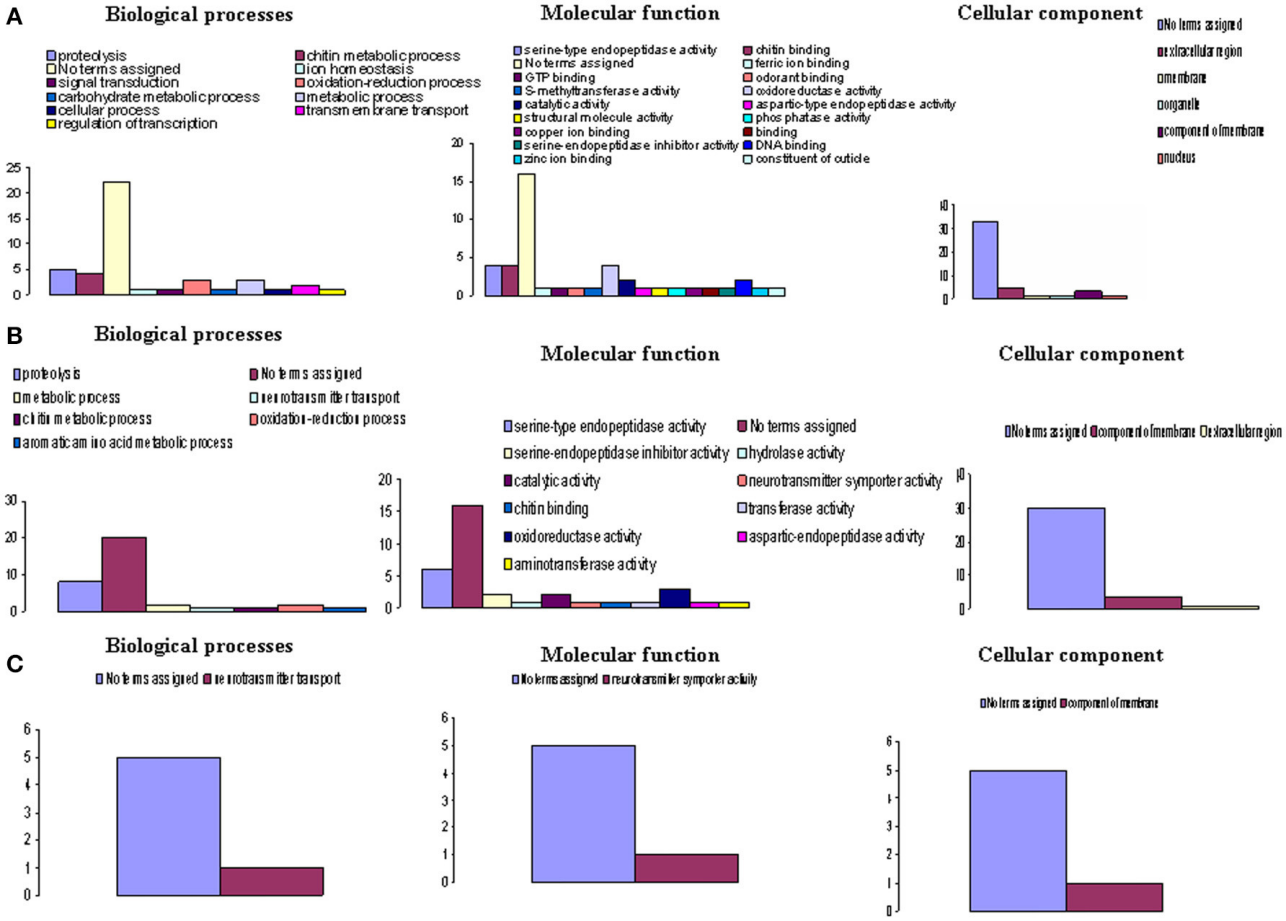
**Functional classes of Gmm genes heterologous to highly differentially expressed Gpg genes**. Highly differentially expressed genes (log_2_ FC > +3 or log_2_ FC < –3) were observed in **(A)**. Tbg stimulated vs. non-stimulated Gpg flies (day-3 sampling); **(B)** Tbg infected vs. non-infected Gpg flies (day-10 sampling); and **(C)** Tbg infected vs. non-infected Gpg flies (day-20 sampling). The X-axis designates the Gene Ontology (GO) category, while the Y-axis provides the number of genes in each GO category.

Table 3 also provides the functional annotation data for each gene at each sampling time. To obtain an overview of the functional groups and categories, we used the GO assignment to classify the functions of the unigenes. According to this process the genes expressed at high levels were classified into three GO groups (Figure 3) and further subdivided into categories: biological process (14 categories), molecular functions (22 categories), and cellular component (6 categories). The category “No terms assigned” was predominant across all GO groups at any investigated time.

### Comparing Gpg gene annotation on the Gmm genome and on a previously used panel of genomes

The global and detailed results of this comparative approach are presented in Supplementary Table S4. Table 4, which is a refined list of Supplementary Table S4, focuses on the expression of Gmm genes that are similar to Gpg genes previously identified as differentially expressed in response to Tbg infection. The results indicate that a high number of Gpg DEGs have orthologs in the Gmm genome. Furthermore, a large number of Gpg (22) and Gmm genes (23) encoding serine proteases were idenetified. Similarly, nine Gpg and nine Gmm genes were identified as encoding chitin binding proteins. Finally, whereas 14 Gmm genes encoding a “Major Facilitator Superfamily transporter” were identified, only one such gene was characterized in Gpg.

**Table 4:** **Identification of Gmm gene orthologs of Gpg genes on the basis of their expression products**.

***Glossina palpalis gambiensis* genes**	**Best hit description-/-name of the encoded proteins**	***Glossina morsitans morsitans* genes**
GLOS_ARP3.1.1	Actin-related protein [*Drosophila melanogaster*]	
	Actin-related protein	GMOY008458
GLOS_DVIR_GJ17549.1.1	Acyltransferase—GJ17549 [*Drosophila virilis*]	
	Acyl-CoA N-acyltransferase	GMOY003123
GLOS_LOC101462532.1.1	Adenylosuccinate lyase-like [*Ceratitis capitata*]	
	Adenylosuccinase	GMOY002461
GLOS_LOC101450467.1.1	Alkaline phosphatase-like—membrane-bound [*Ceratitis capitata*]	
	Alkaline phosphatase	GMOY000067
GLOS_LOC101455841.1.3	Alpha-2-macroglobuline—CD109 antigen-like isoform X5 [*C. capitata*]	
	Alpha-2-macroglobulin	GMOY010996
GLOS_LOC101461571.2.2	Aspartic protease-like (lysosomal) [*Ceratitis capitata*]	
	Aspartic peptidase	GMOY005345; GMOY010103
GLOS_DVIR_GJ18228.1.1; GLOS_FDL.1.2	Beta-hexosaminidase—GJ18228 [*Drosophila virilis*/*D. melanogaster*]	
	Beta-hexosaminidase domain 2-like	GMOY001794
GLOS_KCC2A.2.2	Ca2+/calmodulin-dependent protein kinase type II	
	Ca2+/calmodulin-dependent protein kinase	GMOY006719
GLOS_CEC.2.2; GLOS_CECC.1.1; GLOS_CG10252.2.2	Cecropin [*G. m. morsitans*/*D. yakuba*/*D. melanogaster*]	
	Cecropin (anti-microbial peptide)	GMOY011562; GMOY011563
GLOS_DANA_GF24496.1.1; GLOS_DANA_GF24494.3.12	Chitin binding—GF24496 [*Drosophila ananassae*]	
GLOS_DGRI_GH11353.5.6; GLOS_DGRI_GH14440.1.1	Chitin binding—GH11353 [*Drosophila grimshawi*]	
GLOS_DMOJ_GI10981.2.2; GLOS_DMOJ_GI13574.3.3	Chitin binding—GI10981 [*Drosophila mojavensis*]	
GLOS_DWIL_GK11657.1.1; GLOS_DWIL_GK13541.1.5	Chitin binding—GK11657 [*Drosophila willistoni*]	
GLOS_DPER_GL15114.1.3	Chitin binding—GL15114 [*Drosophila persimilis*]	
	Chitin binding	GMOY002708; GMOY003840; GMOY005251; GMOY005278;
		GMOY009806; GMOY009807; GMOY011054; GMOY011809
		GMOY011810
GLOS_LOC101462140.1.1	Chitinase 3-like [*Ceratitis capitata*]	
	Chitinase-like protein Idgf5	GMOY009161
GLOS_CP305.1.2; GLOS_C4AC3.1.1; GLOS_CP6G1.1.1;	Cytochrome P450 305a1 [*D. melanogaster*]	
GLOS_CP9F2.9.9; GLOS_CP6W1.1.1	Cytochrome P450 9f2 [*D. melanogaster*]	
	cytochrome P450-4g1	GMOY001150; GMOY001939; GMOY006475; GMOY006761
	Cytochrome P450	GMOY002598; GMOY002627; GMOY005461; GMOY007064;
		GMOY007181; GMOY007270; GMOY007652;
		GMOY009767; GMOY009909
GLOS_RN181.1.1	E3 ubiquitin-protein ligase RNF181 homolog	
	E3 ubiquitin-protein ligase SINA like	GMOY002938; GMOY008903
GLOS_ELP2.1.1	Elongator complex protein 2; D. m. GN = Elp2 PE = 1 SV = 1	
	Elongase 4; 9	GMOY008821; GMOY003354
GLOS_LOC101461359.1.1	Endoplasmic reticulum metallopeptidase 1-like isoform X1 [*C. capitata*]	
	Endoplasmic reticulum metallopeptidase 1	GMOY009845; GMOY010241
GLOS_LOC101454791.1.1	Enkurin-like [*Ceratitis capitata*]	
	Enkurin	GMOY009600
GLOS_LOC101459623.1.5	Exonuclease 3′-5′ domain-containing protein 2-like [*C. capitata*]	
	Exonuclease	GMOY012368
GLOS_LOC101459395.1.1	Gamma-glutamyl hydrolase-like [*Ceratitis capitata*]	
	Gamma-glutamyl hydrolase	GMOY000946
GLOS_GSTT1.2.5; GLOS_GST.1.1	Glutathione S-transferase 1-1 [*L. cuprina*/*Musca domestica*]	
	Glutathione S-transferase	GMOY002000; GMOY009373
GLOS_LOC101449625.1.1	Glyoxalase domain-containing protein 4-like isoform X1 [*C. capitata*]	
	Glyoxylase	GMOY008525
GLOS_DVIR_GJ19325.1.1	G-protein receptor activity—GJ19325 [*Drosophila virilis*]	
	G-protein coupled receptor	GMOY009447
GLOS_DWIL_GK15016.1.2	Haemolynph juvenile hormone binding- GK15016- [*D. willistoni*]	
	Haemolymph juvenile hormone binding	GMOY004364
GLOS_H12.1.1	Histone H1.2 [*Drosophila virilis*]	
	Histone H1	GMOY002746
GLOS_IMDH.2.2	Inosine-5′-monophosphate dehydrogenase [*D. melanogaster*]	
	Inosine-5′-monophosphate dehydrogenase	GMOY006458
GLOS_LECA.10.13	Lectin subunit alpha [*Sarcophaga peregrina*]	
	Lectin-like C-type	GMOY001011; GMOY009274
GLOS_LRRX1.1.4	Leucine-rich repeat-containing protein— [*D. discoideum*]	
	Leucine rich repeat containing protein	GMOY010344
GLOS_LSD1.1.1	Lipid storage droplets surface-binding protein 1	
	lipid storage droplet-1	GMOY007510
GLOS_DWIL_GK13707.1.1	Lipid transporter—GK13707 [*Drosophila willistoni*]	
	Lipid transport protein	GMOY002410; GMOY005442; GMOY005442;
GLOS_LOC101449088.1.1	lysM—peptidoglycan-binding domain-containing protein 1-like [*C. capitata*]	
	LysM domain	GMOY008891
GLOS_DPER_GL12526.1.1	Major Facilitator Superfamily-type transporter / — [*D. persimilis*]	
	Major Facilitator Superfamily transporter	GMOY001742; GMOY003428; GMOY003490; GMOY003491;
		GMOY003839; GMOY004738; GMOY005103; GMOY005106;
		GMOY005109; GMOY005501; GMOY007545; GMOY007627;
		GMOY012075; GMOY012352
GLOS_LOC101462556.1.1	MD-2-related lipid-recognition protein-like [*Ceratitis capitata*]	
	MD-2-related lipid-recognition domain	GMOY006406
GLOS_MYSN.1.1	Myosin heavy chain, non-muscle [*D. melanogaster*]	
	Myosin heavy chain	GMOY007533; GMOY008852
GLOS_DMOJ_GI24301.1.1	Neuropeptide Y receptor—GI24301 [*Drosophila mojavensis*]	
	NeuroPeptide Y like receptor / mammalian (Putative)	GMOY011997; GMOY012052
GLOS_DGRI_GH13991.1.1; GLOS_DVIR_GJ10540.1.1	Odorant binding—GH13991 [*Drosophila grimshawi*]/*Drosophila virilis*	
GLOS_OB99B.1.1	Odorant-binding protein 99b	
	Odorant binding protein 1; 2; 7	GMOY000890; GMOY002825; GMOY005548;
	Odorant binding protein 21; 22	GMOY006418; GMOY001476
GLOS_LOC101453268.1.1	Period circadian protein-like [*Ceratitis capitata*]	
	Period circadian protein	GMOY012110
GLOS_LOC101458811.1.1	Pyridoxal kinase-like [*Ceratitis capitata*]	
	Pyridoxal phosphate-dependent transferase	GMOY005488; GMOY005934
		
GLOS_DMOJ_GI20119.1.1; GLOS_DMOJ_GI16517.2.2;	Serine proteases (see details in Supplementary Table S4)	GMOY000672; GMOY002036; GMOY002729; GMOY003271
GLOS_DSEC_GM17695.1.1; GLOS_LOC101456159.4.4;		GMOY003273; GMOY003280; GMOY003693; GMOY003994
GLOS_DMOJ_GI18413.1.2; GLOS_DANA_GF15448.2.3;		GMOY006266; GMOY006369; GMOY006991; GMOY008468
GLOS_AAEL_AAEL007969.1.1; GLOS_LOC101461009.2.2;		GMOY008469; GMOY008958; GMOY008962; GMOY008964
GLOS_EAST.2.2; GLOS_LOC101457953.1.5;		GMOY008965; GMOY008966; GMOY009418; GMOY009436
GLOS_LOC101462986.1.1; GLOS_DWIL_GK19454.1.1		GMOY009757; GMOY010502; GMOY010768
GLOS_LOC101459895.9.9; GLOS_DWIL_GK24139.1.1;		
GLOS_LOC101455430.10.10; GLOS_LOC101455604.4.10;		
GLOS_DMOJ_GI21244.4.5; GLOS_DMOJ_GI24442.1.1;		
GLOS_DVIR_GJ21497.1.3; GLOS_DVIR_GJ22718.8.10;		
GLOS_DVIR_GJ21498.1.1; GLOS_DVIR_GJ21499.1.1;		
GLOS_DMOJ_GI19420.1.1; GLOS_DVIR_GJ17584.1.1	Serine protease inhibitor (Serpin) GI19420 [*D. mojavensis*/*D. virilis*]	
GLOS_DANA_GF14653.1.2; GLOS_DERE_GG24413.1.1;	Serine-type endopeptidase inhibitor— [*D. ananassae*/*D. erecta*]	
GLOS_DWIL_GK10999.1.1; GLOS_LOC101459846.1.2	Serine-type endopeptidase inhibitor /Metalloendopeptidase [*D. willistoni*]	
	Serine proteinase inhibitors (Kazal domain)	GMOY010058
GLOS_DWIL_GK15974.5.7; GLOS_DMOJ_GI22128.1.1	Single domain von Willebrand factor type C [*D. willistoni*]	
	Single domain Von Willebrand factor type C	GMOY003774; GMOY005237; GMOY007078; GMOY010675
GLOS_DWIL_GK22031.1.1	Sulfate transmembrane transporter—GK22031 [*Drosophila willistoni*]	
	Sulfate transporter	GMOY000550
GLOS_LOC101448839.1.1; GLOS_LOC101454308.1.2	Timeless-like isoform X1 protein (circadian rhythm regulation) [*C. capitata*]	
	Timeless protein	GMOY006112
GLOS_TRF.1.1	Transferrin [*Sarcophaga peregrina*]	
	Transferrin family, iron binding site	GMOY004228
GLOS_LOC101463325.1.1	Trypsin-like [*Ceratitis capitata*]	
	Trypsin-like cysteine/serine peptidase domain	GMOY002535; GMOY008308
GLOS_DWIL_GK18237.1.1	UDP-glucuronosyl/UDP-glucosyltransferase—GK18237 [*D. willistoni*]	
	UDP-glucuronosyl/UDP-glucosyltransferase	GMOY007046
GLOS_LOC101451574.1.1	WD repeat-containing protein 81-like isoform X1 [*Ceratitis capitata*]	
	WD40/YVTN repeat-like-containing domain	GMOY010092
GLOS_LOC101458997.1.1	Yellow-like protein (protein of the gelly) [*Ceratitis capitata*]	
	yolk protein 3	GMOY006227

*The table compares the results of the previous Gpg DEG annotation on a panel of various genomes with the corresponding annotation on the Gmm genome*.

*A comparison is presented between the Gpg and the Gmm genes on the basis of the identified proteins they encode*.

### Homologies between identified Gmm genes that are heterologous to Gpg DEGs with genes from other organisms

**Figure 4:**
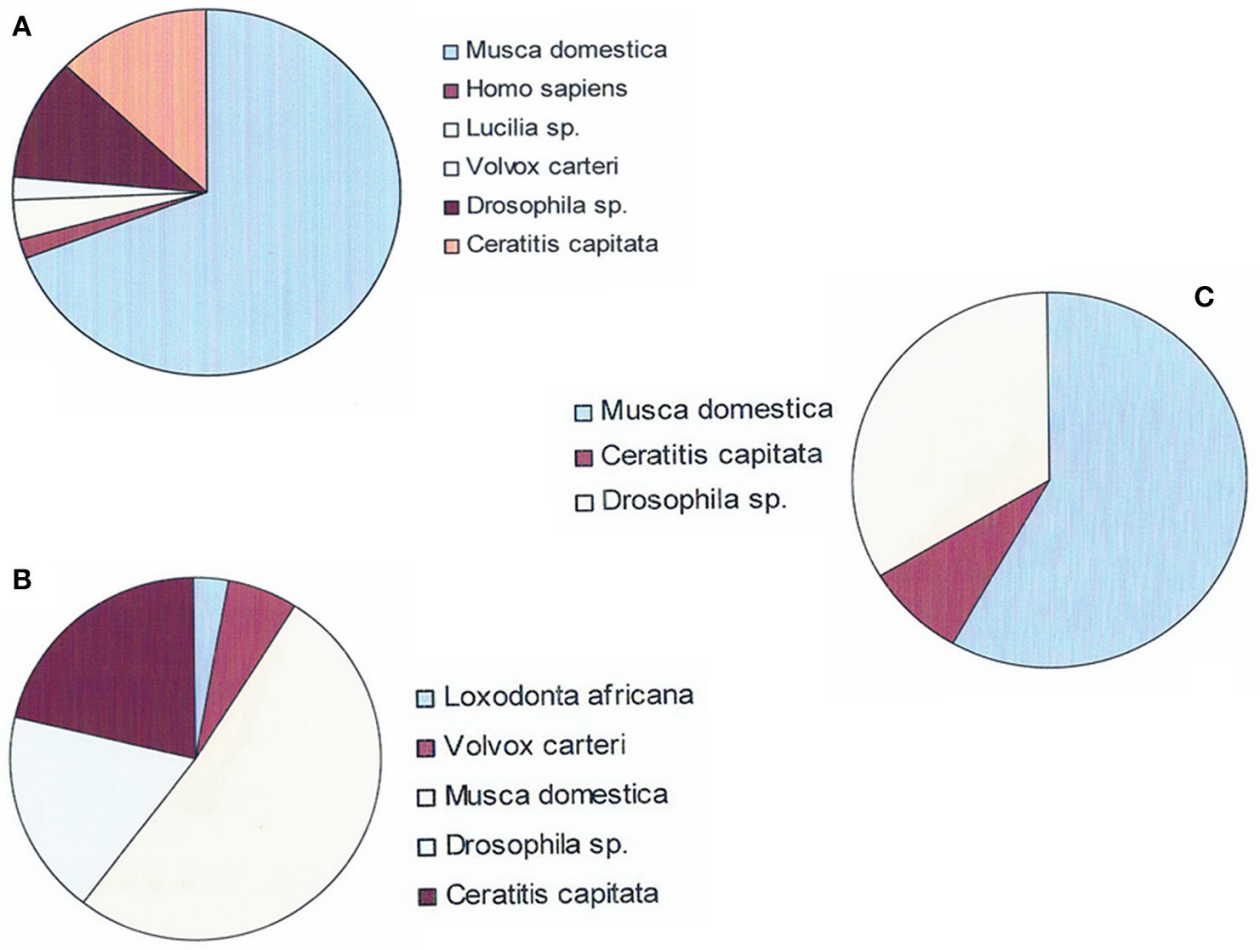
**Species on which Gmm genes were mapped in order to characterize genes previously annotated as “hypothetical.” (A)** Mapping of the 284 Gmm genes heterologous to Gpg DEGs in trypanosome stimulated vs. non-stimulated flies (day-3 sampling). **(B)** Mapping of the 139 Gmm genes heterologous to Gpg DEGs in trypanosome infected vs. non-infected flies (day-10 sampling). **(C)** Mapping of the 59 Gmm genes heterologous to Gpg DEGs in trypanosome infected vs. non-infected flies (day-20 sampling).

In order to identify genes previously annotated as “uncharacterized” or “hypothetical,” we used the BLASTx program to identify heterologous genes among various organisms listed in the NCBI databases. Homologies with a cut-off *E* < 10^−5^ and-/-or displaying the highest hits score were selected; the minimum accepted homology level was 60%. Table 5 presents the results of the recorded annotation, and Figure 4 presents the species from which genomes the genes to be annotated displayed the best match.

**Table 5:** **Gmm gene heterologs of Gpg DEGs matching genes from other organism databases**.

**Genes**	**log_2_ FC**	**Homology with other organisms (>60%)**
GMOY000550	−0.90	*Musca domestica* sodium-independent sulfate anion transporter-like (LOC101893700), mRNA
GMOY001137	1.09	*Musca domestica* aminomethyltransferase, mitochondrial-like (LOC101895864), mRNA
GMOY001742	-0.9	*Ceratitis capitata* synaptic vesicle glycoprotein 2B-like (LOC101452461), transcript variant X3, mRNA
GMOY002004	−1.78	*Musca domestica* putative fatty acyl-CoA reductase CG5065-like (LOC101898308), mRNA
GMOY002004	1.8	*Musca domestica* putative fatty acyl-CoA reductase CG5065-like (LOC101898308), mRNA
GMOY002024	0.96	*Musca domestica* phosphotriesterase-related protein-like (LOC101890186), mRNA
GMOY002356	2.31	*C. capitata* CUGBP Elav-like family member 2-like (LOC101455154), transcript variant X1 to X3, mRNA
GMOY002461	0.82	*Musca domestica* adenylosuccinate lyase-like (LOC101900029), mRNA
GMOY002486	2.6	*Musca domestica* potassium channel subfamily K member 9-like (LOC101895107), mRNA
GMOY002535	1.5	*Ceratitis capitata* serine protease easter-like (LOC101451852), mRNA
GMOY002729	1.91	*Lucilia sericata* clone LScDNA1 putative salivary trypsin mRNA, complete cds
GMOY002729	3.0	*Musca domestica* serine proteinase stubble-like (LOC101890358), mRNA
GMOY002938	−1.10	*Musca domestica* uncharacterized LOC101893009 (LOC101893009), mRNA
GMOY003158	2.70	*Musca domestica* uncharacterized LOC101895341 (LOC101895341), mRNA
GMOY003161	1.44	*Musca domestica* thyrotropin receptor-like (LOC101887582), mRNA
GMOY003354	2.48	*Ceratitis capitata* elongation of very long chain fatty acids protein AAEL008004-like (LOC101449680), mRNA
GMOY003354	6.3	*Musca domestica* elongation of very long chain fatty acids protein AAEL008004-like (LOC101893043), mRNA
GMOY003443	1.27	*Musca domestica* uncharacterized LOC101889318 (LOC101889318), partial mRNA
GMOY003590	1.77	*Musca domestica* collagen alpha-1(IV) chain-like (LOC101897761), transcript variant X3, mRNA
GMOY003590	1.3	*Musca domestica* collagen alpha-1(IV) chain-like (LOC101897761), transcript variant X3, mRNA and variant X1, X2
GMOY003830	2.75	*Homo sapiens* BAC clone CH17-465I15 from chromosome unknown, complete sequence (= hypothetical)
GMOY003830	3.7	*Volvox carteri f, nagariensis* mRNA for pherophorin-dz1 protein
GMOY003839	0.95	*Musca domestica* putative inorganic phosphate cotransporter-like (LOC101889974), mRNA
GMOY003949	1.01	*M. domestica* glutamine-fructose-6-phosphate aminotransferase [isomerizing] 2-like (LOC101889985), transcript
GMOY004309	2.44	*Musca domestica* fatty acid synthase-like (LOC101893120), mRNA
GMOY004332	2.1	*Drosophila willistoni* GK20732 (Dwil\GK20732), mRNA /Fatty acyl-CoA reductase
GMOY004337	5.8	*D. willistoni* GK20950 (Dwil\GK20950), mRNA Bardet-Biedl syndrome 4 protein homolog (= hypothetical on Gmm genome)
GMOY004337	6.6	*Ceratitis capitata* Bardet-Biedl syndrome 4 protein homolog (LOC101449311), mRNA (= hypothetical on Gmm genome)
GMOY004589	1.09	*Musca domestica* muscle M-line assembly protein unc-89-like (LOC101890868) mRNA
GMOY004712	1.65	*Musca domestica* acyl-protein thioesterase 1-like (LOC101890399), mRNA
GMOY004738	−0.78	*Musca domestica* facilitated trehalose transporter Tret1-like (LOC101891733), transcript variant X1, Mrna
GMOY004873	1.6	*Musca domestica* transmembrane and TPR repeat-containing protein CG4341-like (LOC101893859), mRNA
GMOY005102	6.28	*Musca domestica* N-acetylgalactosaminyltransferase 4-like (LOC101894376), mRNA
GMOY005106	−1.22	*Drosophila willistoni* GK13266 (Dwil\GK13266), mRNA / Major facilitator superfamily transporter
GMOY005278	2.93	*Musca domestica* mucin-5AC-like (LOC101899868), mRNA
GMOY005278	1.9	*Musca domestica* mucin-5AC-like (LOC101899868), mRNA
GMOY005345	-6.5	*Musca domestica* lysosomal aspartic protease-like (LOC101894831), mRNA
GMOY005487	3.48	*Musca domestica* LIM/homeobox protein Lhx4-like (LOC101900654), mRNA
GMOY005488	1.81	*Musca domestica* alpha-methyldopa hypersensitive protein-like (LOC101888467), mRNA
GMOY005527	0.87	*Musca domestica* c-1-tetrahydrofolate synthase, cytoplasmic-like (LOC101891351), transcript variant X2, mRNA
GMOY005606	6.3	*Musca domestica* leucine-rich repeat-containing protein 15-like (LOC101899894), mRNA (= hypothetical on Gmm genome)
GMOY005934	2.72	*Ceratitis capitata* cysteine sulfinic acid decarboxylase-like (LOC101455610), mRNA
GMOY006111	1.1	*Drosophila willistoni* GK14673 (Dwil\GK14673), mRNA (Gonadal trypsine)
GMOY006205	1.2	*Ceratitis capitata* DNA replication licensing factor Mcm5-like (LOC101458261), mRNA
GMOY006406	1.69	*Musca domestica* ecdysteroid-regulated 16 kDa protein-like (LOC101898283), mRNA
GMOY006406	1.7	*Musca domestica* ecdysteroid-regulated 16 kDa protein-like (LOC101898283), mRNA
GMOY006458	−0.78	*Musca domestica* inosine-5′-monophosphate dehydrogenase-like (LOC101895820), mRNA
GMOY006671	4.0	*Musca domestica* uncharacterized LOC101890025 (LOC101890025), mRNA
GMOY006761	1.99	*Musca domestica* cytochrome P450 CYP4G13v2 mRNA, complete cds
GMOY006761	2.4	*Musca domestica* cytochrome P450 CYP4G13v2 mRNA, complete cds
GMOY006761	-2.1	*Musca domestica* cytochrome P450 CYP4G13v2 mRNA, complete cds
GMOY006875	-2.0	*Musca domestica* membrane-bound alkaline phosphatase-like (LOC101896753), mRNA
GMOY006875	-1.8	*Musca domestica* membrane-bound alkaline phosphatase-like (LOC101896753), mRNA
GMOY006979	1.08	*Musca domestica* phosrestin-2-like (LOC101892743), Mrna
GMOY007046	2.0	*Ceratitis capitata* UDP-glucuronosyltransferase 2B13-like (LOC101462823), transcript variant X2, mRNA
GMOY007131	0.97	*Musca domestica* inositol-3-phosphate synthase-like (LOC101889622), mRNA
GMOY007148	2.10	*Ceratitis capitata* fatty acid synthase-like (LOC101463409), mRNA
GMOY007497	6.3	*Musca domestica* NADH-cytochrome b5 reductase 3-like (LOC101897795), transcript variant X2, mRNA
GMOY007523	1.44	*Musca domestica* collagen alpha-1(IV) chain-like (LOC101895032), transcript variant X3, mRNA
GMOY007523	1.1	*Musca domestica* collagen alpha-1(IV) chain-like (LOC101895032), transcript variant X3, mRNA
GMOY007560	-1.0	*C. capitata* polypeptide N-acetylgalactosaminyltransferase 2-like (LOC101448408), mRNA (= hypothetical on Gmm genome)
GMOY007584	1.1	*Ceratitis capitata* synaptotagmin-1-like (LOC101450559), mRNA (= hypothetical on Gmm genome)
GMOY008017	1.88	*Volvox carteri f. nagariensis* mRNA for pherophorin-dz1 protein (= hypothetical when mapped on Gmm genome)
GMOY008017	1.2	*Volvox carteri f, nagariensis* mRNA for pherophorin-dz1 protein (= hypothetical when mapped on Gmm genome)
GMOY008266	-1.3	*Drosophila willistoni* GK24772 organic anion transporter (Dwil\GK24772), mRNA
GMOY008308	1.9	*Drosophila melanogaster* easter (ea), transcript variant A, mRNA
GMOY008458	5.74	*Musca domestica* actin, indirect flight muscle-like (LOC101895248), mRNA
GMOY008525	0.97	*Drosophila willistoni* GK21885 (Dwil\GK21885), mRNA
GMOY008601	2.58	*Musca domestica* fatty acid synthase-like (LOC101893120), mRNA
GMOY008601	4.1	*Drosophila willistoni* GK12914 (Dwil\GK12914), mRNA
GMOY008602	2.02	*Drosophila pseudoobscura pseudoobscura* GA26263 (Dpse\GA26263), mRNA
GMOY008602	-2.5	*Drosophila willistoni* GK12914 (Dwil\GK12914), mRNA
GMOY008852	0.93	*Musca domestica* myosin heavy chain, non-muscle-like (LOC101892851), transcript variant X1 to X3, Mrna
GMOY008966	4.4	*Loxodonta africana* kallikrein-11-like (LOC100667195), mRNA
GMOY008973	0.80	*Lucilia cuprina* alpha esterase (LcaE7) mRNA, implicated in organophosphate resistance, complete cds
GMOY009018	1.59	*Ceratitis capitata* uncharacterized LOC101448539 (LOC101448539), transcript variant
GMOY009018	1.8	*Musca domestica* uncharacterized LOC101899326 (LOC101899326), mRNA
GMOY009079	1.23	*Musca domestica* fatty acid synthase-like (LOC101893120), mRNA
GMOY009375	7.56	*Musca domestica* uncharacterized LOC101900740 (LOC101900740), mRNA
GMOY009394	−5.56	*Musca domestica* CCAAT/enhancer-binding protein-like (LOC101898926), mRNA
GMOY009447	−1.22	*Ceratitis capitata* calcitonin gene-related peptide type 1 receptor-like (LOC101462563), mRNA
GMOY009600	1.20	*Musca domestica* enkurin-like (LOC101897351), mRNA
GMOY009845	0.6	*Musca domestica* endoplasmic reticulum metallopeptidase 1-like (LOC101898765), transcript variant X3, mRNA
GMOY009903	2.75	*M. domestica* strain rspin nicotinic acetylcholine receptor beta 3 subunit (nAChRbeta3) gene, nAChRbeta3-C allele, complete cds
GMOY009983	1.7	*Drosophila grimshawi* GH17190 (Dgri\GH17190), mRNA
GMOY010224	6.9	*Musca domestica* uncharacterized LOC101889990 (LOC101889990), partial mRNA
GMOY010224	3.6	*Musca domestica* uncharacterized LOC101889990 (LOC101889990), partial mRNA **(** = > Hypothetical**)**
GMOY010241	0.8	*Musca domestica* endoplasmic reticulum metallopeptidase 1-like (LOC101898765), transcript variant X3, mRNA
GMOY010481	−1.53	*Musca domestica* protein Wnt-5-like (LOC101892275), mRNA
GMOY010972	0.73	*Musca domestica* phenoloxidase subunit A3-like (LOC101897997), transcript variant X1 and X2, mRNA
GMOY011232	1.32	*Drosophila melanogaster* PAPS synthetase (Papss), transcript variant A, mRNA variant A to H
GMOY011418	0.87	*Drosophila willistoni* GK13980 (Dwil\GK13980), mRNA / glycogen synthase
GMOY011618	-0.9	*Ceratitis capitata* putative fatty acyl-CoA reductase CG5065-like (LOC101456246), transcript variant X2, mRNA
GMOY012052	3.4	*Drosophila pseudoobscura pseudoobscura* GA30114 Neuropeptide Y (Dpse\GA30114), mRNA
GMOY012069	9.1	*Musca domestica* uncharacterized LOC101891108 (LOC101891108), mRNA
GMOY012069	5.3	*Musca domestica* uncharacterized LOC101891108 (LOC101891108), mRNA
GMOY012075	1.56	*Drosophila melanogaster* CG31663 (CG31663), transcript variant B, mRNA (Major facilitator superfamily transporter)
GMOY012075	-1.7	*Drosophila willistoni* GK15555 (Dwil\GK15555), mRNA (Major facilitator superfamily transporter)
GMOY012352	0.94	*Ceratitis capitata* monocarboxylate transporter 10-like (LOC101448353), transcript variant X1, mRNA

*Black fonts, day-3 samples; 3952a4Blue fonts, day-10 samples; ee1f25Red fonts, day-20 samples*.

Among the 284 Gmm genes heterologous to the day-3 Gpg DEGs samples, 54 genes showed significant matches with other organisms in the investigated databases. The top homology matches were *Drosophila* sp. (11.1%), *Ceratitis capitata* (13%), and *Musca domestica* (68.5%). The remaining 7.4% of genes matched with either *Homo sapiens* (1.8%*), Lucilia sericata* (3.5%), or *Volvox carteri* (2.1%). Similarly, among the 139 Gmm genes heterologous to the day-10 Gpg DEGs samples, 33 genes showed significant matches with other organisms. The top homology matches were *Drosophila* (18.2%), *Ceratitis capitata* (21.3%), and *Musca domestica* (51.5%). The remaining 9% of genes matched with either *Loxodonta africana* (2.9%) or *Volvox carteri* (6.1%). Finally, among the 59 DEGs from day-20 samples, 12 DEGs displayed significant matches with *Musca domestica* (58.7%), *Ceratitis capitata* (8.3%), or *Drosophila* sp (33%).

Several trends appear when comparing results from the annotation reported in Table 5 with those reported in Supplementary Tables S1–S3 (or in Table 3, regarding the genes in which the differential expression level was –2 < log_2_ FC or log_2_ FC > 2). First, many genes were not annotated; second, for genes that were annotated, the fold-change was identical; and finally, several genes that were annotated as “Hypothetical” when mapped on the Gmm genome could be identified when mapped on other databases. This was the case regarding the genes GMOY003830 (i.e., = > Pherophorin-dz1 protein, when annotated on *Volvox carteri*), GMOY004337 (i.e., = > Bardet –Biedl syndrome 4 protein homolog, when annotated on *D. williston*i or *Ceratitis capitata*), GMOY005606 (i.e., = > leucine-rich repeat-containing protein 15-like, when annotated on *Musca domestica*), GMOY007560 (i.e., = > N-acetylgalactosaminyl transferase 2-like, when annotated on *C. capitata*), GMOY007584 (i.e., = > Synaptotagmin-1-like, when annotated on *C capitata*), and GMOY008070 (i.e., = > Pherophorin-dz1 protein, when annotated on *Volvox carteri*).

## Discussion

The chronic and acute forms of sleeping sickness endemic to sub-Saharan Africa are caused by two *Trypanosoma* sub-species, Tbg and Tbr, which are, respectively, transmitted to their vertebrate hosts by the *Glossina* species Gpg and Gmm (Aksoy et al., 2014; Beschin et al., 2014). Nevertheless, the biological cycles, vertebrate transmission processes, and pathogenicity development of the two parasites are similar. Recently, in the context of an anti-vector strategy project to fight the disease, we performed a global transcriptomic analysis of Gpg gene expression associated with fly infection by Tbg. More precisely, we attempted to characterize genes that were differentially expressed according to the status of the fly at several sampling times (i.e., non-infected, infected, or self-cured). This included genes that could be involved in the fly's vector competence, and consequently genes that could possibly be manipulated in order to reduce or even suppress this competence.

The similarities between the Tbg and Tbr life cycles prompted us to determine whether the Gmm genome carried genes that could be heterologous to the Gpg DEGs, which could then allow the development of common molecular approaches. Accordingly, the Gpg sequences resulting from the previous RNA-seq *de novo* assembly (Hamidou Soumana et al., 2015) were mapped on the Gmm genome, the DEGs were characterized, and the corresponding genes were annotated.

When the Gpg sequences were mapped and annotated on a panel of various databases (*C. capitata, D. melanogaster, D. willistoni, D. virilis, D. mojavensis, Acyrthosiphon pisum, Hydra magnipapillata, Anopheles* sp., *Bombyx* sp., *Aedes* sp., and *G. morsitans*; Hamidou Soumana et al., 2015) we identified 553 (S vs. NS), 52 (I10 vs. NI10), and 143 (I20 vs. NI20) DEGs. In contrast, we identified 284 (S vs. NS), 139 (I10 vs. NI10) and 59 (I20 vs. NI20) DEGs when sequences were mapped and annotated on the *G. m. morsitans* database (using its whole genome annotated on the *Drosophila melanogaster, Aedes aegypti, Anopheles gambiae, Culex quinquefasciatus*, and *Phlebotomus papatasi* databases; International Glossina Genome Initiative, 2014). The differences in the number of identified DEGs, as well as the high number of “uncharacterized” genes, could be due to differences in the database panels used to annotate Gpg or Gmm. We cannot exclude the possibility that some of the Gpg DEGs do not have heterologous genes in Gmm, or that some of them could be specific to either Gpg or Gmm and consequently cannot be annotated yet. Nevertheless, regarding I10 vs. NI10 sampling (and in contrast to the two other experimental conditions), the number of recorded DEGs was more than 2-fold higher when the Gpg transcripts were mapped on the Gmm genome, prompting questions of how this is possible. However, at this stage of our research we cannot offer a satisfactory explanation.

We examined the potential influence of database panel composition by annotating the Gmm DEGs on a separate set of databases that included *D. melanogaster* as an internal control. The results (Table 5) clearly demonstrate the validity of the annotation process, since all Gmm genes (GMOY, etc.) were annotated (best hit description and fold-change) on the novel set of databases as they had been annotated on the former set (Table 3), and that several genes could be identified thanks to their annotation primarily on the *Volvox carteri* or *Musca domestica* databases which had never been used before, and despite the fact these organisms (algae and mouse) are genetically distant from the tsetse fly.

The most important observation regarding our objective is that almost all of the Gpg genes previously considered to be potentially involved in tsetse fly vector competence (cf. Hamidou Soumana et al., 2015) had a “countrepart” (i.e., heterologous genes) in the Gmm genome, despite the fact that none of the Gpg DEGs matched with any Gmm genes. This was the case for the large array of genes encoding peptidases, especially serine peptidases (represented by more than 20 genes), identified in the genomes of both fly species. This was similarly observed for ~10 genes present in both genomes that encode chitin binding proteins, since chitin metabolism is involved in the ability of tsetse flies to host trypanosomes (Maudlin and Welburn, 1994; Welburn and Maudlin, 1999), in addition to cecropin (an antimicrobial peptide), among others (Weiss et al., 2014).

Here, we were particularly interested in detecting the presence or not of genes with a reported role in the immunity of tsetse flies or other organisms (Weiss et al., 2014). Genes encoding Pro3 protein (GMOY009756, GMOY000672) and transferrin (GMOY004228) were identified. Pro3 has a potential function as a serine protease (tyrosinase) and is specifically produced by the proventriculus, an organ that plays an important role in the tsetse immune response. This protein could be involved in the immune response via activation of the cascade of prophenol oxidase and melanization (Jiang et al., 1998). Moreover, the gene GMOY0010488 was identified as encoding an “immuno reactive putative protease inhibitor” that is overexpressed in trypanosome stimulated or infected Gpg flies. The transferrin gene was overexpressed in both stimulated and infected Gpg flies; this result is in agreement with Geiser and Winzerling (2012), who reported on the role of transferrin in the immune response of insects, as well as its role in iron transport. By reducing the oxidative stress in tsetse fly guts, transferrin may promote the survival of trypanosomes. Guz et al. (2012) observed transferrin overexpression after challenge with bacteria, even at a higher level than what is typically observed in the case of infection by trypanosomes.

The gene GMOY011809 encodes Pro 1 peritrophin, which is a constituent of the peritrophic membrane (PM). The PM is established after the fly takes its first blood meal, and it is permanently renewed by the proventriculus (Moloo et al., 1970; Tellam et al., 1999). The PM primarily functions to envelop the blood meal and protect the intestinal epithelium against abrasion by ingested matter, although it can also represent an obstacle to the passage of ingested parasites into the ectoperitrophic space (Lehane, 1997; Hegedus et al., 2009). The gene GMOY005278 encodes mucin, which participates with peritrophin in the composition of the PM.

We have also identified genes encoding antimicrobial peptides: in Supplementary Table S1, GMOY01052 through GMOY010524 encode attacin, whereas GMOY0011562 and GMOY0011563 encode cecropin. Furthermore, both attacin and cecropin are overexpressed in Gpg trypanosome stimulated or infected flies.

Our work is the first comparison of its kind between the two *Glossina* species. This is primarily due to the fact that the different scientific teams working on HAT commonly focus on investigating either Gmm (and the acute form of trypanosomiasis) or Gpg (and the chronic form of trypanosomiasis), but not both together. Indeed, one of our most relevant findings is the observation that Gmm has the same genes at its disposal that Gpg may use to control its vector competence. Importantly, this comparison will assist future studies in revealing common molecular targets to increase the refractoriness of either fly species to infection by trypanosomes.

## Author contributions

Conceived and designed the experiments: IH, AG. Performed the experiments: IH, BT, SR, HP. Analyzed the data: IH, SR, HP, AG. Wrote the paper: AG.

### Conflict of interest statement

The authors declare that the research was conducted in the absence of any commercial or financial relationships that could be construed as a potential conflict of interest. The reviewer CGFdL and handling Editor declared their shared affiliation, and the handling Editor states that the process nevertheless met the standards of a fair and objective review.
